# Neuromuscular recruitment strategies of the vastus lateralis according to sex

**DOI:** 10.1111/apha.13803

**Published:** 2022-03-02

**Authors:** Yuxiao Guo, Eleanor J. Jones, Thomas B. Inns, Isabel A. Ely, Daniel W. Stashuk, Daniel J. Wilkinson, Kenneth Smith, Jessica Piasecki, Bethan E. Phillips, Philip J. Atherton, Mathew Piasecki

**Affiliations:** ^1^ 6123 Centre of Metabolism Ageing & Physiology (COMAP) MRC‐Versus Arthritis Centre for Musculoskeletal Ageing Research and National Institute for Health Research (NIHR) Nottingham Biomedical Research Centre School of Medicine University of Nottingham Derby UK; ^2^ 8430 Department of Systems Design Engineering University of Waterloo Waterloo Ontario Canada; ^3^ 6122 Musculoskeletal Physiology Research Group Sport, Health and Performance Enhancement Research Centre Nottingham Trent University Nottingham UK

**Keywords:** motor unit, neuromuscular junction, sex differences, vastus lateralis

## Abstract

**Aim:**

Despite males typically exhibiting greater muscle strength and fatigability than females, it remains unclear if there are sex‐based differences in neuromuscular recruitment strategies e.g. recruitment and modulation of motor unit firing rate (MU FR) at normalized forces and during progressive increases in force.

**Methods:**

The study includes 29 healthy male and 31 healthy female participants (18‐35 years). Intramuscular electromyography (iEMG) was used to record individual motor unit potentials (MUPs) and near‐fibre MUPs from the vastus lateralis (VL) during 10% and 25% maximum isometric voluntary contractions (MVC), and spike‐triggered averaging was used to obtain motor unit number estimates (MUNE) of the VL.

**Results:**

Males exhibited greater muscle strength (*P* < .001) and size (*P* < .001) than females, with no difference in force steadiness at 10% or 25% MVC. Females had 8.4% and 6.5% higher FR at 10% and 25% MVC, respectively (both *P* < .03), while the MUP area was 33% smaller in females at 10% MVC (*P* < .02) and 26% smaller at 25% MVC (*P* = .062). However, both sexes showed similar increases in MU size and FR when moving from low‐ to mid‐level contractions. There were no sex differences in any near‐fibre MUP parameters or in MUNE.

**Conclusion:**

In the vastus lateralis, females produce muscle force via different neuromuscular recruitment strategies to males which is characterized by smaller MUs discharging at higher rates. However, similar strategies are employed to increase force production from low‐ to mid‐level contractions. These findings of similar proportional increases between sexes support the use of mixed sex cohorts in studies of this nature.

## 1 INTRODUCTION

Skeletal muscle contraction is regulated by the coordinated activation of motoneurons and muscle fibres. The fundamental neuromuscular element regulating muscle contraction is the motor unit (MU), consisting of a motor neuron and the muscle fibres it innervates.^1^ Increases in muscle force are largely mediated by two neuromuscular recruitment strategies, the recruitment of additional, progressively larger MUs, and an increase in MU firing rate (FR), referred to as MU recruitment and rate modulation, respectively.^2^ Several studies have highlighted adaptative remodelling of MUs structure and function in response to exercise training, ageing and disease,^3^, ^4^, ^5^, ^6^ which may influence recruitment strategies, however the majority of data are only available in males.

Males generally possess greater muscle strength than females in upper and lower extremities, which is largely explained by greater muscle size.^7^ Conversely, although task‐specific, females are generally more resistant to neuromuscular fatigue when assessed at a normalized contraction level,^8^ which in the knee extensors, is likely explained by differing fibre type ratios with a 7%‐23% greater proportion of type I fibres in vastus lateralis (VL) in females.^9^, ^10^ Sex differences of the hormonal milieu also influence neuromuscular function; testosterone and oestrogen are the major sex hormones in males and females, respectively, and each exhibits a range of neuroprotective effects in motoneurons, such as dendritic maintenance and axonal sprouting.^11^ Furthermore, hormonal metabolites are associated with the release of brain‐derived neurotrophic factors (BDNF),^12^ which are key mediators of synaptic plasticity.^13^ Acutely, differences in sex hormones partly explain the variability in fatigability in females across phases of the menstrual cycle.^14^ Such differences in the hormonal milieu are difficult to experimentally control for and may explain why females are often underrepresented in studies of neuromuscular physiology.^15^

Surface electromyography (sEMG) has been commonly applied to study sex‐based differences of neuromuscular function and recruitment strategies.^16^, ^17^ However, such approaches are limited by the distance between activated MUs and recording electrodes,^18^ offering poor quality signals in females due to the greater subcutaneous tissues,^19^ and in some cases being influenced by adjacent muscles.^20^ These limitations can be overcome with the use of intramuscular EMG (iEMG), which also has the added benefit of revealing further electrophysiological parameters relevant to MU size and complexity.^21^ Although we have previously reported the sex‐based divergent trajectory of MU FR from middle to older age in long‐term trained master athletes,^22^ comparisons of normative values in healthy young males and females at differing contraction levels are unknown. The aims of the present study were to compare individual MU properties and neuromuscular recruitment strategies, as well as the MU number estimates (MUNE) in the VL of healthy young males and females. We hypothesized motor unit size and firing rate would differ at normalized contraction levels, with no sex‐based differences in recruitment strategies when moving from a low‐ to a mid‐level contraction.

## 2 RESULTS

**FIGURE 1:**
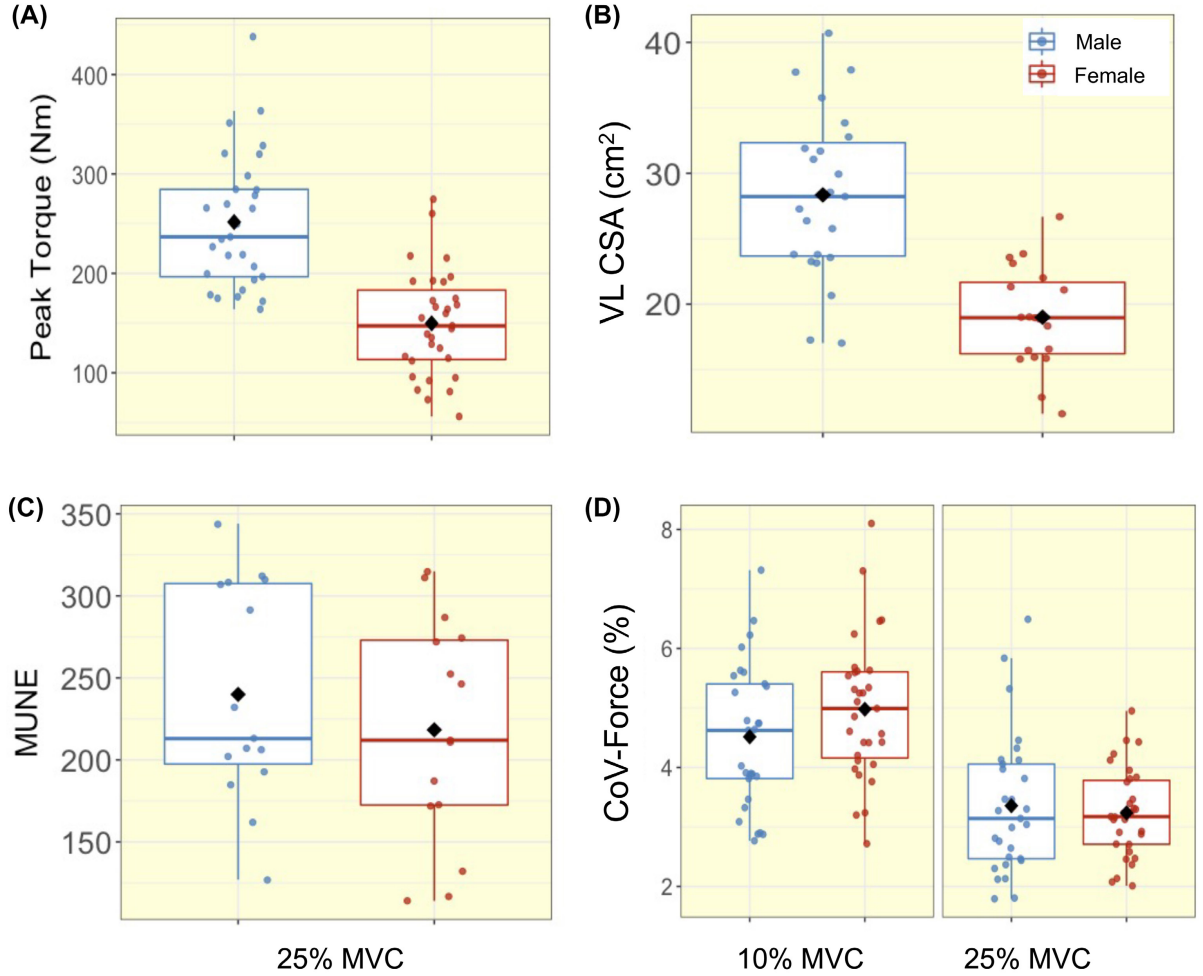
Box‐and‐jitter plots of the individual participant means, and group means (black dot) of (A) peak torque, (B) vastus lateralis cross‐sectional area, (C) motor unit number estimates (MUNE), and (D) force steadiness at 10% and 25% maximum voluntary contraction (MVC), in males (blue) and females (red). CoV, coefficient of variation

The means and standard deviations for the participant's characteristics are given in Table 1. Significant differences between males and females were detected for weight, height, BMI, peak torque and VL CSA (all *P* < .05). There was no significant sex difference for age (*P* = .49). There was a significant interaction between sex and contraction level in force steadiness (*P* = .008). Both males and females showed greater improvement in force control from low‐ to mid‐level contractions (both *P* < .001) with females exhibiting a slightly greater decrease in force fluctuations compared with males. Individual values are shown in Figure 1.

**TABLE 1:** Participant characteristics

Measure	Males (n = 29)	Females (n = 31)	*P* value
Age (years)	23.7 (5.0)	22.9 (3.6)	.490
Weight (kg)	81.2 (11.6)	63.1 (10.1)	**<.001**
Height (cm)	180.3 (7.5)	165.8 (6.8)	**<.001**
BMI (kg/m^2^)	25.0 (2.9)	23.0 (3.2)	.**012**
Peak Torque (Nm)	251.65 (67.72)	158.95 (46.69)	**<.001**
VL CSA (cm^2^)	28.35 (6.43)	19.00 (3.90)	**<.001**

Note

Data are reported as mean (standard deviation). Values in bold reflect statistically significant (*P* < .05) results.

Abbreviations: BMI, body mass index; VL CSA, vastus lateralis cross‐sectional area.

**FIGURE 2:**
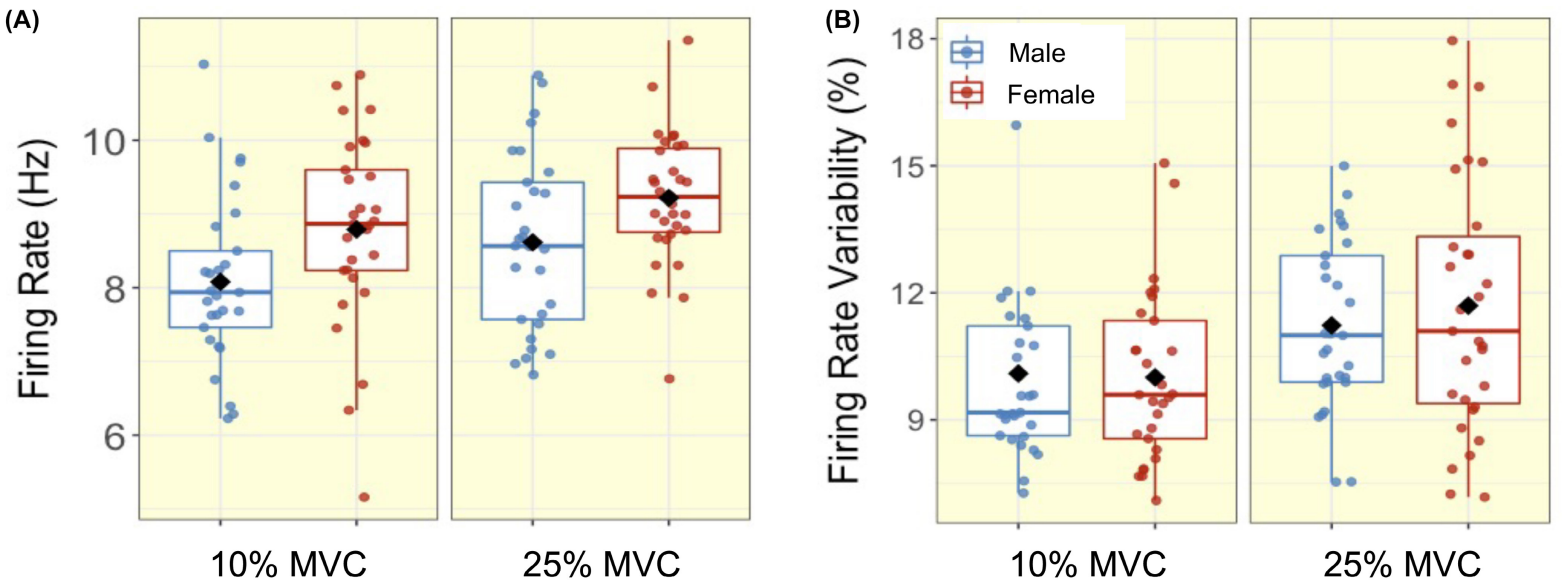
Box‐and‐jitter plots of the individual participant means, and group means (black dot) of (A) motor unit (MU) firing rate and (B) firing rate variability in males (blue) and females (red) at 10% and 25% maximum voluntary contraction (MVC)

**FIGURE 3:**
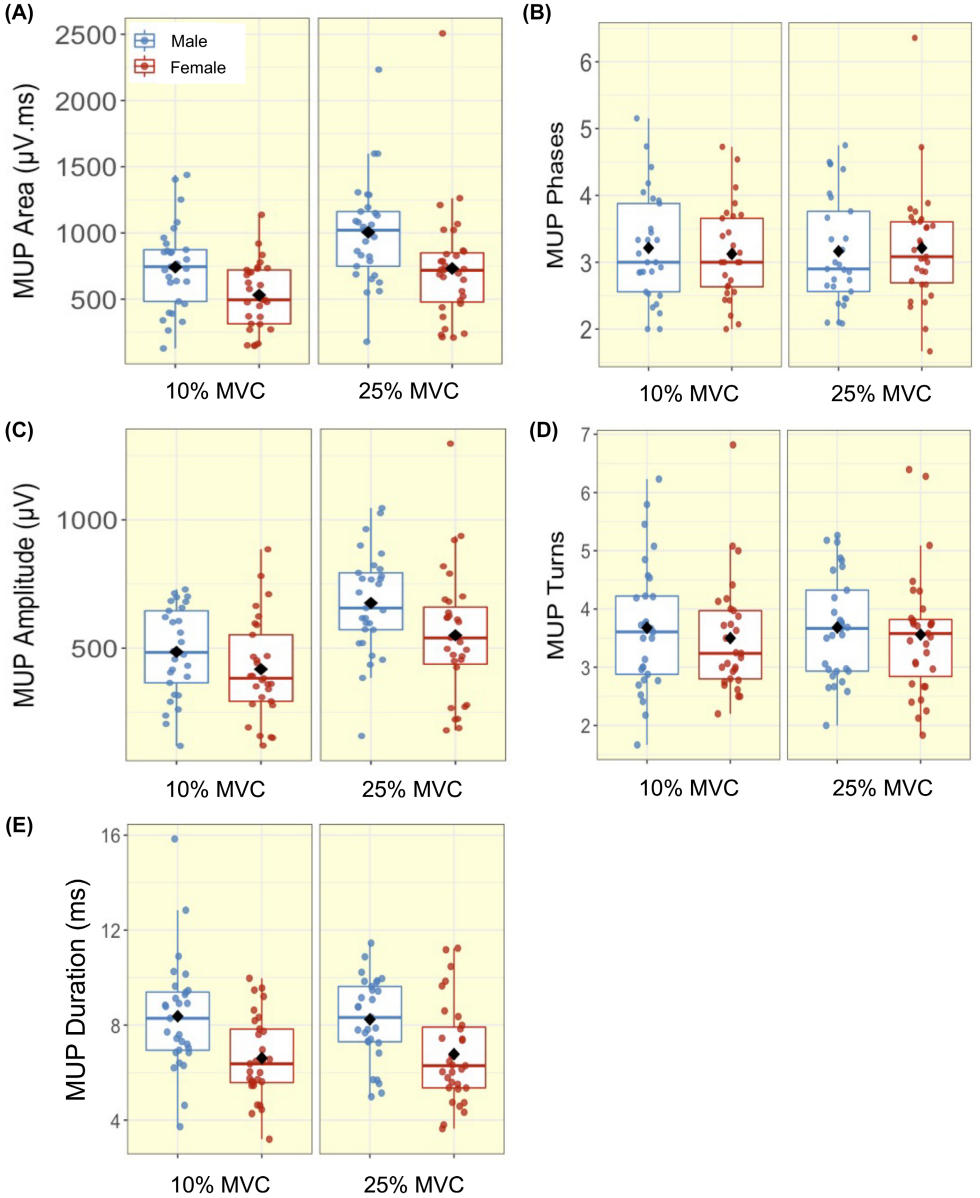
Box‐and‐jitter plots of the individual participant means, and group means (black dot) of motor unit potential (MUP) (A) Area; (B) Phases; (C) Amplitude; (D) Turns; (E) Duration in males (blue) and females (red) at 10% and 25% maximum voluntary contraction (MVC)

**FIGURE 4:**
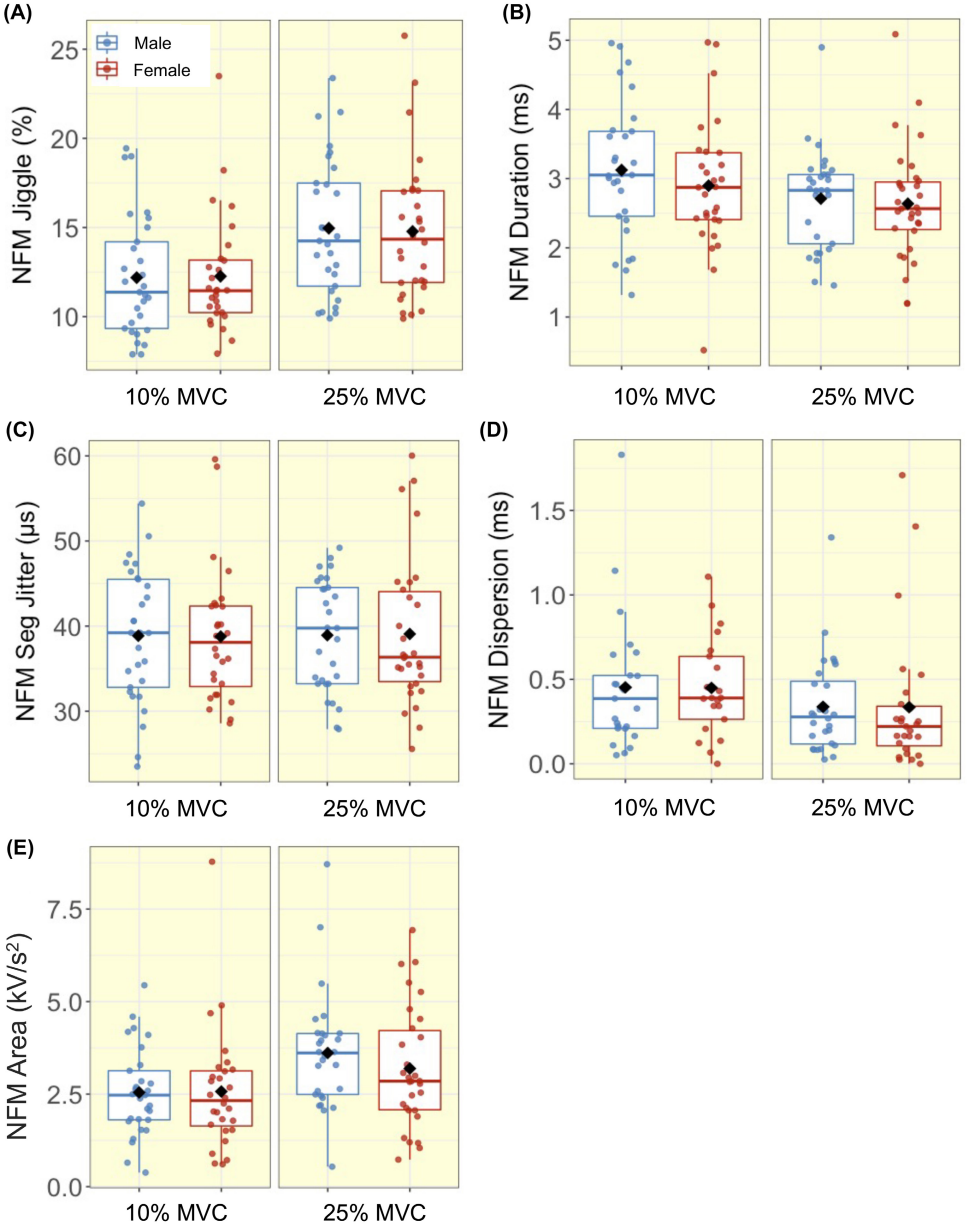
Box‐and‐jitter plots of the individual participant means, and group means (black dot) of near fibre motor unit potential (NFM) (A) Jiggle; (B) Duration; (C) Segment (Seg) Jitter; (D) Dispersion; (E) Area in males (blue) and females (red) at 10% and 25% maximum voluntary contraction (MVC)

At 10% MVC, the mean number of MUs isolated per person was 26 in males with a mean of 6 MUs per sampling position, and 17 in females with 4 MUs per sampling position. At 25% MVC, the mean number of MUs isolated per person was 31 in males with 8 MUs per sampling position and, 22 in females with 6 MUs per sampling position. A total of 1645 MUPs were inspected and included for analysis in males, and 1207 in females. Individual mean values for all functional, MU and NFM parameters are shown in Figures 1, 2, 3, 4. There were no significant interactions between sex and contraction level in any of the MU parameters. When interactions were removed from the model, multilevel linear regression revealed females had greater MU FR at both 10% (mean; M: 8.08 Hz; F: 8.79 Hz) and 25% (M: 8.62 Hz; F: 9.20 Hz) MVC (both *P* < .05, Table 2, Figure 2A). No sex‐based differences (*P* > .10) were detected in MU FR variability at either contraction level (Table 2, Figure 2B). MUP area was smaller in females at 10% (M: 741 μV·ms; F: 531 μV·ms) (*P* = .006), with a non‐significant trend at 25% MVC (M: 1005 μV·ms; F:775 μV·ms) (*P* = .062). MUP duration was shorter at 10% (M: 8.37 ms; F: 6.61 ms) and 25% MVC (M: 8.24 ms; F: 6.84 ms) in females when compared with males (both *P* < .01). There were no significant sex‐based differences in any other MU characteristic (Table 2, Figure 3).

**TABLE 2:** Motor unit properties in different sexes

Parameter	Level	Beta	95%CI	*P* value
MU FR (Hz)	10% MVC	0.73	0.14 to 1.32	.**018**
	25% MVC	0.61	0.07 to 1.14	.**031**
MU FR variability (%)	10% MVC	0.26	−0.74 to 1.25	.617
	25% MVC	0.49	−0.72 to 1.70	.433
MUP area (μV·ms)	10% MVC	−210.08	−352.90 to −67.27	.**006**
	25% MVC	−215.41	−436.82 to 6.00	.062
MUP phases	10% MVC	−0.07	−0.46 to 0.31	.707
	25% MVC	0.05	−0.38 to 0.48	.823
MUP amplitude (μV)	10% MVC	−62.83	−151.27 to 25.61	.170
	25% MVC	−92.01	−206.31 to 22.29	.120
MUP turns	10% MVC	−0.19	−0.73 to 0.35	.500
	25% MVC	−0.10	−0.60 to 0.40	.691
MUP duration (ms)	10% MVC	−1.85	−2.93 to −0.77	.**001**
	25% MVC	−1.35	−2.33 to 0.37	.**009**
NFM Jiggle (%)	10% MVC	−0.06	−1.84 to 1.72	.947
	25% MVC	0.09	−1.85 to 2.03	.928
NFM duration (ms)	10% MVC	−0.16	−0.60 to 0.29	.486
	25% MVC	−0.02	−0.40 to 0.36	.926
NFM Seg Jitter (μs)	10% MVC	−0.22	−4.29 to 3.84	.915
	25% MVC	0.47	−3.42 to 4.36	.814
NFM dispersion (ms)	10% MVC	0.08	−0.22 to 0.38	.622
	25% MVC	0.03	−0.25 to 0.32	.825
NFM area (kV/s^2^)	10% MVC	0.001	−0.71 to 0.71	.998
	25% MVC	−0.22	−0.97 to 0.54	.575

Note

Beta value and 95% confidence interval (CI) represent the model predicted change per unit from males to females, shown separately for 10 and 25% maximum voluntary contraction (MVC). All statistical analysis was based on multilevel mixed effect linear regression models with each subject as an independent cluster. The values in bold reflect statistically significant (*P* < .05) results.

Abbreviations: FR, firing rate; MU, motor unit; MUP, motor unit potential; NFM, near fibre motor unit potential; Seg, segment.

**FIGURE 5:**
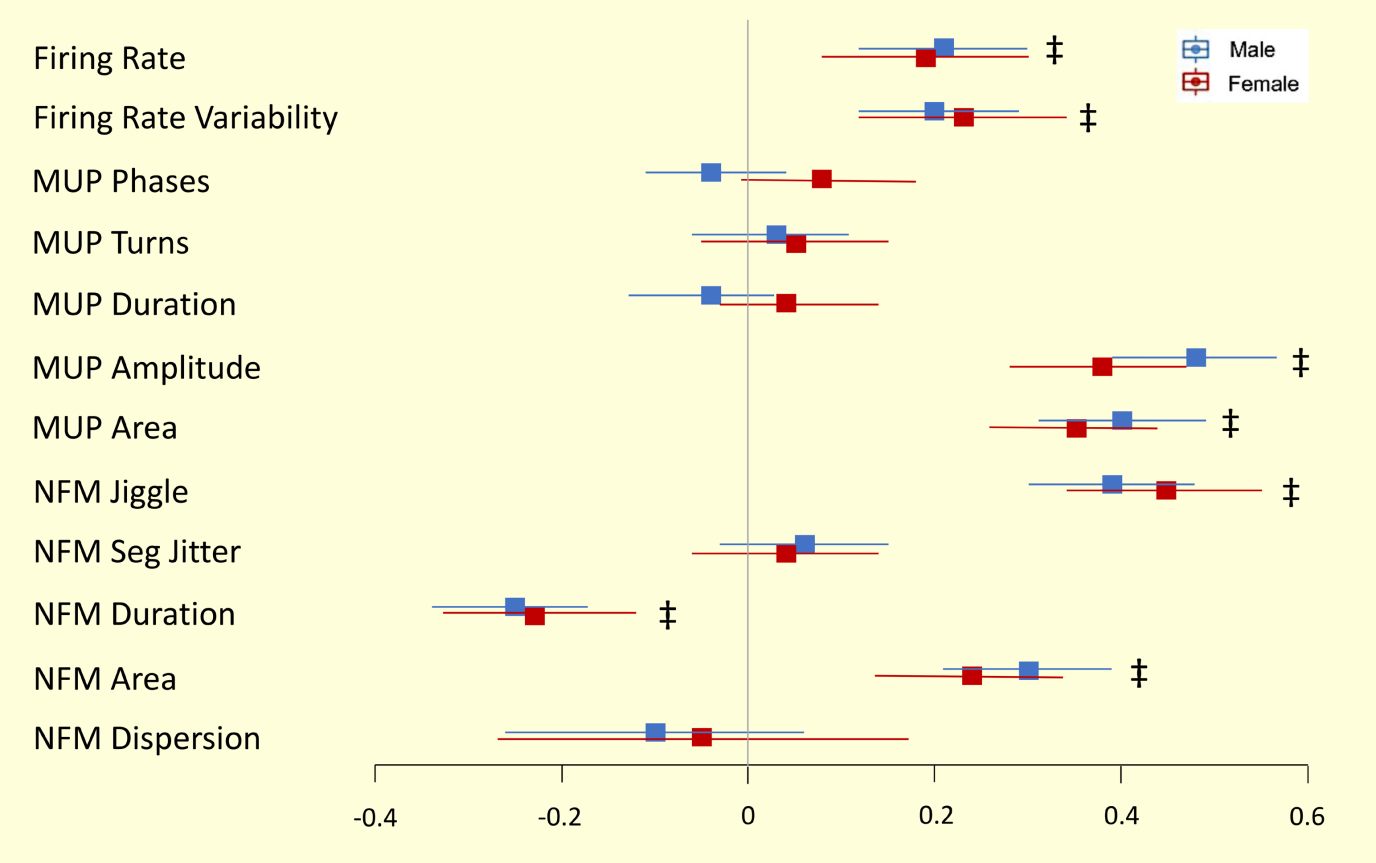
Forest plots of the standardized regression coefficient estimate for associations between motor unit characteristics and contraction levels in males and females models. Beta value and 95% confidence intervals (CI) represent the standardized model predicted change per unit from 10% to 25% maximum voluntary contraction (MVC). All statistical analysis was based on multilevel mixed effect linear regression models with each subject as an independent cluster. Standardized values of each parameter make the comparisons executable between men and women. ^‡^ *P* < .001

With increasing contraction level, both males and females exhibited higher MU FR and MU FR variability, as well as greater MUP amplitude and larger MUP area (all *P* ≤ .001, Table 3, Figures 2 and 3). NFM area and NFM jiggle were greater, and NFM duration was shorter, with the higher contraction level, differing to a similar extent in males and females (all *P* < .001, Table 3, Figure 5). There were no interactions between sex and contraction level in any of the MU parameters, indicating the difference from 10% to 25% MVC did not differ between males and females (Figure 5).

**TABLE 3:** Motor unit properties at different contraction levels

Parameter	Sex	Beta	95%CI	*P* value
MU FR (Hz)	Males	0.50	0.28 to 0.71	**<.001**
	Females	0.43	0.18 to 0.69	.**001**
MU FR variability (%)	Males	0.99	0.52 to 1.45	**<.001**
	Females	1.18	0.63 to 1.72	**<.001**
MUP area (μV·ms)	Males	273.62	215.81 to 331.43	**<.001**
	Females	199.35	148.13 to 250.56	**<.001**
MUP phases	Males	−0.05	−0.14 to 0.04	.302
	Females	0.10	−0.02 to 0.21	.098
MUP amplitude (μV)	Males	188.21	153.75 to 222.67	**<.001**
	Females	142.40	106.27 to 178.53	**<.001**
MUP turns	Males	0.05	−0.11 to 0.20	.565
	Females	0.09	−0.08 to 0.26	.316
MUP duration (ms)	Males	−0.16	−0.52 to 0.20	.381
	Females	0.13	−0.16 to 0.42	.371
NFM Jiggle (%)	Males	3.08	2.38 to 3.79	**<.001**
	Females	3.08	2.34 to 3.82	**<.001**
NFM duration (ms)	Males	−0.40	−0.54 to −0.26	**<.001**
	Females	−0.34	−0.50 to −0.18	**<.001**
NFM Seg Jitter (μs)	Males	0.77	−0.37 to 1.91	.188
	Females	0.51	−0.85 to 1.87	.463
NFM dispersion (ms)	Males	−0.09	−0.23 to 0.05	.228
	Females	−0.05	−0.28 to 0.18	.655
NFM area (kV/s^2^)	Males	0.98	0.68 to 1.29	**<.001**
	Females	0.72	0.39 to 1.05	**<.001**

Note

Beta value and 95% confidence interval (CI) represent the model predicted change per unit from 10% to 25% maximum voluntary contraction (MVC), shown separately for males and females. All statistical analysis was based on multilevel mixed effect linear regression models with each subject as an independent cluster. The values in bold reflect statistically significant (*P* < .05) results.

Abbreviations: FR, firing rate; MU, motor unit; MUP, motor unit potential; NFM, near fibre motor unit potential; Seg, segment.

## 3 DISCUSSION

This is the first study to compare neuromuscular recruitment strategies and motor unit number estimates (MUNE) of the VL using iEMG techniques in healthy young males and females. Despite males being stronger and having larger muscles, there were no differences in force steadiness at either low‐ or mid‐level contraction levels between sexes. At each contraction level assessed, females displayed smaller markers of MU size and greater MU FR, indicating differing recruitment strategies to achieve a normalized force. When assessing the difference between contraction levels, both males and females exhibited higher MU FR and greater MUP size, which differed to a similar extent in both sexes, indicating a similar recruitment strategy to generate proportional increases in force. In addition, there was no significant sex‐based differences in MUNE of the VL. These data reveal divergent neuromuscular recruitment strategies between sexes to achieve a normalized force, which follow similar trajectories with increasing force.

Consistent with previous studies, females exhibited 39% smaller muscle size (CSA of the VL), which was reflected in a 37% lower strength.^7^, ^23^, ^24^ The greater MU FR of females shown here in VL is in an agreement with some, but not all, previously published data and again highlights probable muscle‐specific confounders. For instance, females exhibited higher MU FR and MU FR variability compared with males in elbow flexors, flexor digitorum indicis, biceps, knee extensors and tibialis anterior.^25^, ^26^, ^27^, ^28^ However, others reported no sex difference in knee extensors during 30% MVC^29^ and significantly greater MU FR at 100% MVC in tibialis anterior in males.^30^, ^31^ As expected, in the current study MU FR increased with increasing force levels to a similar extent in both males and females, accompanied by greater MU FR variability. Although differing at each contraction level, the similar proportional increase in MU FR in males and females indicates both sexes follow similar discharge pattern increases from low to mid‐level normalized contractions.

Despite large differences in muscle strength, force steadiness—representing the ability to hold a constant force, which is also influenced by MU FR and its variability^25^, ^32^, ^33^, ^34^—did not differ between sexes at either contraction level. Differing from the current findings, Inglis^28^ found that females had a greater MU FR variability and greater fluctuation in steadiness than males during dorsiflexion in tibias anterior muscles, which may indicate a muscle‐specific sex difference. In the current study, both males and females exhibited greater force steadiness at 25% MVC when compared with 10% MVC, consistent with Inglis' finding that very high‐ and low‐level force outputs have greater fluctuations compared with mid‐level force outputs.^28^, ^35^

The size of a MU can be estimated by the size of the MUP recorded using intramuscular electrodes. As previously mentioned, males typically exhibit larger muscle size than females,^9^, ^36^ with increases in force mediated by recruitment of additional larger MUs and increases in MU FR. Here MUP area and duration were smaller in females, which reflect smaller MU size. When viewed alongside the greater MU FR in females, it suggests that at the normalized force levels assessed here, females are more reliant on MU FR than on recruitment of larger MUs, when compared with males. As expected, markers of MU size increased with larger contraction levels, as larger MUs are recruited to produce larger forces. Again, the trajectory of each was similar for males and females, indicating MU recruitment strategies moving between these force levels do not differ between sexes.

A near fibre MUP (NFM) is derived from a MUP, such that is primarily composed of contributions from MU fibres close to the intramuscular electrode.^21^ Here there were no sex differences in any NFM parameters at either contraction level. When comparing 10% MVC and 25% MVC contractions, NFM area increased, while NFM duration decreased, albeit to a similar extent in both sexes. These contractions‐induced alterations may be the result of the activation of larger MU fibres with greater conduction velocity during higher level contractions.^37^, ^38^

Increases in NFM instability, as measured by NFM jiggle or NFM segment jitter, can reflect increases in neuromuscular junction (NMJ) transmission instability with age^22^, ^39^, ^40^, ^41^ and in diabetic neuropathy.^3^ In the current study, NFM instability, as measured by NFM jiggle, increased with contraction level for both sexes, and to similar extents. NFM jiggle is based on variability in the amplitudes of NFM shapes, and although these amplitude changes are normalized by the size of the NFM, it is possible that these increases with contraction level may be due to the recruitment of larger MUs with more MU fibres contributing to larger NFMs at 25% MVC. Combined with the lack of a sex difference in NFM segment jitter, it is clear that NMJ transmission instability in the VL is sensitive to contraction level and is similar in healthy young males and females. However, there were no statistically significant contraction‐based differences in NFM segment jitter, which is based on variability in the occurrence times of NFM segments and is not affected by NFM size, indicating it is less sensitive to the influences of contraction level.

The mean values of MUNE in males (240 ± 66) and females (218 ± 68) reported here are similar to those we have previously reported in male cohorts,^5^, ^41^ and highlight the repeatability of this method in this muscle group when applying identical experimental procedures. Although the MUNE should be viewed as an index relative to the number of MUs within a muscle and not a true anatomical count, the similar values reported here in males and females support minimal sex‐based differences in the number of VL MUs. Additionally, differences in MU size, as reflected by MUP area at 25% MVC, were minimal compared with differences in total muscle size, therefore the current data support the notion that sex‐based differences in total muscle size are largely explained by greater individual fibre size in males.^36^

Although providing a high level of detail of MU structure and function via MUPs and NFMs sampled in deep and superficial muscle regions, regardless of subcutaneous tissue amount, iEMG is sensitive to contraction level and reliably identifying individual MU activity at high levels in this muscle can be problematic. Therefore, data presented here were obtained during low and mid‐level contractions only. Secondly, we did not control for hormonal fluctuations in females naturally occurring during the menstrual cycle nor the use of oral contraceptives, the latter of which may influence vascular tone.^42^ Thirdly, the limb dominance was not assessed and the right leg was uniformly measured across all the participants. This is a direct comparison of MU features during sustained contractions in young males and females and it was not possible to accurately quantify MU recruitment thresholds, which may bias findings if they differ according to sex in the VL. Further investigations concerning neural drive and influence of hormones on neural drive are still required to further understand the sex‐based differences in the motor nervous system.

In summary, when compared with males, females exhibited smaller VL MUs with higher MU FR, when assessed at a single normalized contraction level. However, both males and females showed similar increases in MU size and MU FR from a low‐ to a mid‐level contraction, indicating a similar neuromuscular recruitment strategy. These results suggest that although sex‐based neuromuscular differences are apparent at a single contraction level, relative differences between levels are similar in this widely studied muscle group. These data do not support the notion of excluding females from studies of this nature.

## 4 MATERIALS AND METHODS

### 4.1 Participants

Twenty‐nine healthy male and 31 healthy female participants, aged 18‐35 years, were recruited via advertisement posters in the local community. All the participants volunteered to take part in the studies and provided written informed consent. Prior to enrolment, all participants completed a comprehensive clinical examination, and metabolic screening was conducted at the School of Medicine, Royal Derby Hospital Centre. All participants were recreationally active. Participants with metabolic disease, lower limb musculoskeletal abnormalities, acute cerebrovascular or cardiovascular disease, active malignancy, uncontrolled hypertension, or those on medications that impact muscle protein metabolism or modulate vascular tone were excluded. Stage of the menstrual cycle, or cycle status, was not assessed in the female participants. Methods or type of birth control was not assessed in the female participants.

### 4.2 Anthropometry

Body mass and height were measured using calibrated scales and a stadiometer, respectively, for the calculation of body mass index (BMI). Ultrasound was used to measure the cross‐sectional area of the VL using an ultrasound probe (LA523 probe, B‐mode, frequency range 26‐32 Hz and MyLabTM50 scanner, Esaote, Genoa, Italy) at the anatomical mid‐point of the muscle, which was identified between the greater trochanter and the mid‐point of the patella with participants lying supine. Ultrasound images were acquired aligning the superior edge of the probe following a medial‐to‐lateral direction position on the skin, beginning, and ending the image capture at aponeurosis borders. A water‐based conductive gel was applied on the surface of the ultrasound probe to enhance the fidelity of the image without causing excessive contact pressure on the skin during the acquisition of the images. Images were subsequently analysed using ImageJ software (National Institutes of Health, USA) to quantify CSA measurements. The mean area of three images was taken as CSA. The CSA of eight female participants was measured using magnetic resonance imaging (MRI) with a T1‐weighted turbo 3D sequence on a 0.25‐T G‐Scan (Esaote, Genoa, Italy). Continuous transversal images with a 6‐mm slice were acquired and analysed by using Osirix imaging software (Osirix medical imaging, Osirix, Atlanta, GA, United States) through tracing around the VL following the contour of the aponeurosis. VL CSA values are available for 23 males and 19 females.

### 4.3 Muscle strength and force steadiness

The maximum isometric voluntary contraction force (MVC) of the right knee extensor was assessed with the participants sitting in a custom‐built chair with hips and knees flexed at ~90°. The lower leg was securely attached to a force dynamometer with non‐compliant straps (purpose‐built calibrated strain gauge, RS125 Components Ltd, Corby, UK) slightly above the medial malleolus. Surface adhesive electrodes (detailed below) were placed on the skin. A seat belt was fastened across the pelvis to minimize movement of the upper trunk during the test. To obtain the external knee joint moment arm, the distance from centre of the force strap to the lateral femoral condyle was measured. After a standardized warm‐up of submaximal contractions, participants were instructed to perform each trial with maximal effort, with real‐time visual feedback and verbal encouragement. This was repeated a further two to three times, with 60 seconds rest intervals between each, and if the difference between last two attempts was less than 5%, the highest value, in Newtons was accepted as MVC. Peak torque during the selected MVC was also determined.

**FIGURE 6:**
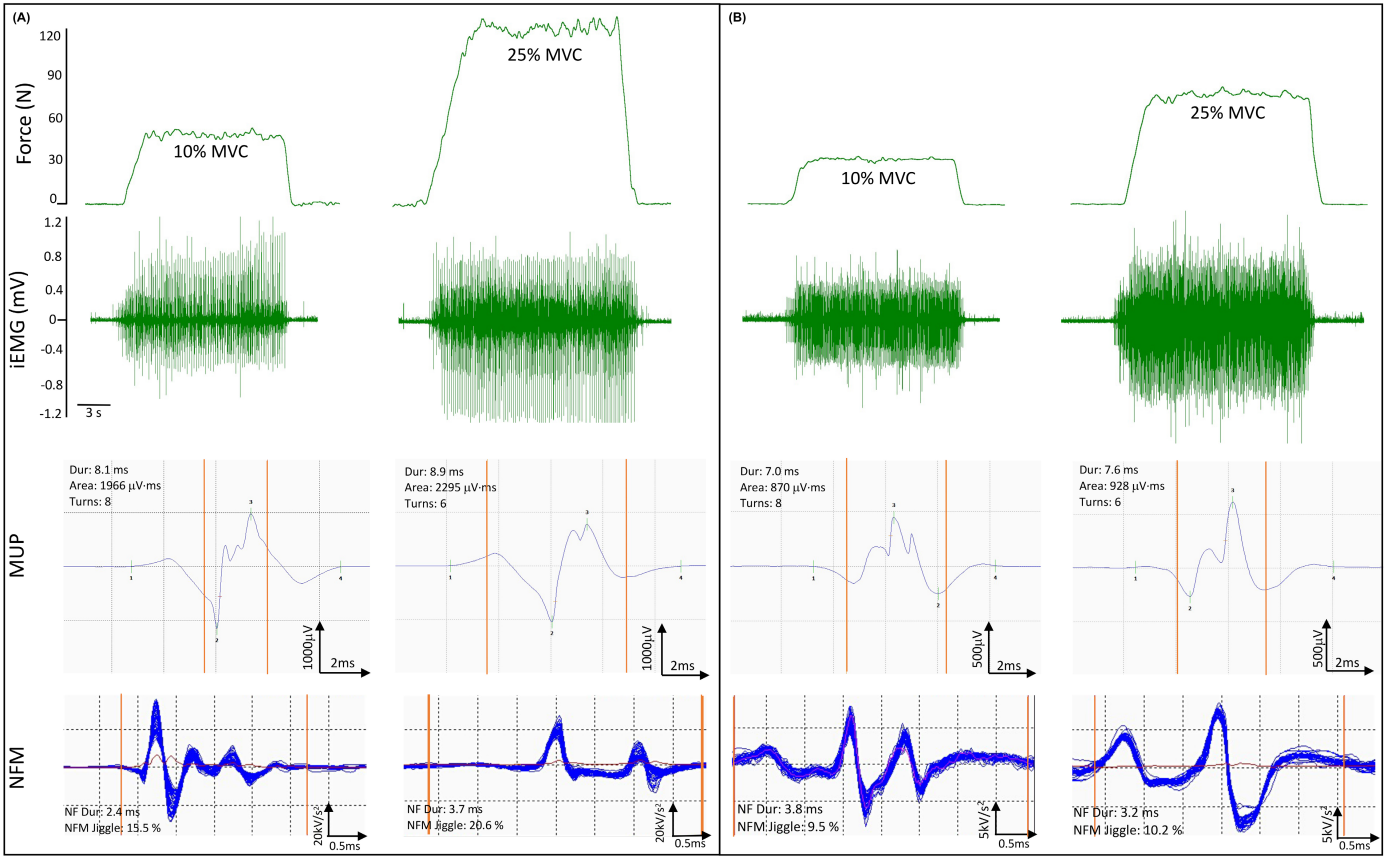
Representative data from a male (A) and a female (B) participant. Top panels show knee extensor force traces at 10% and 25% of MVC, and corresponding intramuscular electromyography (iEMG) raw data recorded from the vastus lateralis. A representative MUP template and corresponding NFM shimmer plot isolated from each contraction are shown below each iEMG signal. Vertical orange lines on MUPs and NFMs indicate the start and end time of the NFM. Dur, duration; kV, kilovolt; ms, millisecond; mV, millivolt; MVC, maximum voluntary contraction; MUP; motor unit potential; N, Newtons; NF, near fibre; NFM, near fibre MUP; μV, microvolt

Prior to needle insertion and multiple sustained contractions, a familiarization trial was performed in which participants were instructed to match the target force at each contraction level for 12‐15 seconds. Following the practice trial, the intramuscular needle electrode was inserted into the mid‐point of the VL, and participants were instructed to perform between four and six sustained isometric contractions at 10% and 25% MVC, each lasting 12‐15 seconds with a target line displayed on the screen and real‐time force feedback (Figure 6). Participants had 20‐30 seconds rest between each contraction. Force steadiness was quantified as the coefficient of variation of the force [CoV; (SD/mean) × 100]. To avoid corrective actions when reaching the target line, the first two passes of the target (<1s) were excluded from the calculation. The mean CoV at each contraction level was calculated from the middle two contractions.

### 4.4 Surface electromyography

An active recording sEMG electrode (disposable self‐ adhering Ag‐AgCl electrodes; 95 mm^2^, Ambu Neuroline, Baltorpbakken, Ballerup, Denmark) was placed over the motor point located around the mid‐point of the VL, identified using a cathode probe (Medserve, Daventry, UK) to apply percutaneous electrical stimulation at 400 V, pulse width of 50 μs and current of around 8 mA (DS7A Digitimer, Welwyn Garden City, Hertfordshire, UK) with a self‐adhesive anode electrode (Dermatrode, Farmadomo, NL) placed over the right gluteus. A reference electrode was placed over the patella tendon and a common ground electrode placed over the patella. The common ground electrode served for both sEMG and iEMG measurements. sEMG signals were sampled at 10kHz, and bandpass filtered between 5 and 5 kHz (1902 amplifier, Cambridge Electronics Design Ltd., Cambridge, UK) and digitized with a CED Micro 1401 data acquisition unit (Cambridge Electronic Design) for offline analysis.

### 4.5 Compound muscle action potential

The compound muscle action potential (CMAP) of the VL was evoked by a manually triggered stimulator (model DS7A; Digitimer) using percutaneous stimulation (Medserve, Daventry, UK) of the proximal femoral nerve (approximately halfway between the anterior superior iliac spine and the pubic tubercle) with a carbon‐rubber anode electrode (Dermatrode self‐adhering electrode, 5.08 cm in diameter; Farmadomo Linde Homecare Benelux Bv, Leiden, The Netherlands) placed over the skin overlying the gluteus muscle. The stimulator voltage was fixed at 400 V and the pulse width at 50 μs, with the current increased incrementally until the M‐wave amplitude plateaued. At this point, the current was increased again by ~30 mA to ensure supramaximal stimulation, ensuring a sharp rise time of the negative peak of the m‐wave.

### 4.6 Intramuscular electromyography

A 25‐mm disposable concentric needle electrode (N53153; Teca, Hawthorne, New York, USA) was inserted at the muscle belly of VL, adjacent to the recording surface electrode over the motor point, to a depth of 1.5‐2 cm depending on the muscle size. The iEMG shared the same ground electrode as the sEMG, which was placed over the patella. iEMG signals were recorded using Spike2 (Version 9.06), sampled at 50 kHz and bandpass filtered at 10 Hz to 10 kHz (1902 amplifier; Cambridge Electronic Design Ltd, Cambridge, UK) and stored for future off‐line analysis.

Prior to EMG and CMAP assessments, participants performed a series of voluntary, low‐level contractions once the needle was positioned to ensure adequate signal‐to‐noise ratio, thus ensuring the recording needle electrode was close to depolarizing fibres. Each participant then performed the sustained voluntary isometric contractions as detailed above (Figure 6). After a 10% and 25% MVC contraction, to avoid repeat sampling of the same MUs, the needle electrode was repositioned by the combinations of twisting the bevel edge 180 degrees and withdrawing by ~5 mm. This process was repeated until four to six contractions from spatially distinct areas (from deep to superficial portions) and recorded.^18^ Participants had ~30 seconds rest between each contraction.

### 4.7 EMG analysis

Decomposition‐based quantitative electromyography (DQEMG) software was used to detect motor unit potentials (MUPs), extract motor unit potential trains (MUPTs) generated by individual MUs from the sustained iEMG signals (ramps excluded) and estimate, via ensemble averaging, their corresponding surface MUPs (sMUPs) from the sEMG signals.^43^ MUPTs that were composed of MUPs from more than one MU or had fewer than 40 MUPs were excluded. The occurrence times of individual MUPs within a MUPT were used to trigger and align sEMG signal epochs for ensemble‐averaging to produce an estimate of their corresponding sMUPs. All MUP and sMUP templates were visually inspected and their markers adjusted, where required, to correspond to the onset of negative phase (sMUP only), end, and positive and negative peaks of the waveforms.

MUP amplitude was measured from the maximal positive and negative peaks, and the MUP area was taken as the total area within the MUP duration (onset to end) and is indicative of MU size. The number of phases and turns are measures of MUP complexity and are classified as the number of components above or below the baseline (phases) and a change in waveform direction of at least 25 μV (turns), which indicates the level of temporal dispersion across individual muscle fibre contributions to a single MUP. MU FR was assessed as the rate of MUP occurrences within a MUPT, expressed as the number of occurrences per second (Hz). MU FR variability is reported as the coefficient of variation (CoV) for the interspike interval (ISI) displayed as a percentage.

A near fibre MUP (NFM) is defined as the acceleration of its corresponding MUP (Figure 6) and calculated by applying a second‐order, low‐pass differentiator to the MUP which effectively reduces the recording area of the needle electrode to within ~350 μm, thereby ensuring only potentials from fibres closest to the needle electrode significantly contribute to the NFM and reducing interference from distant active fibres of other MUs. All NFMs (and corresponding MUPs) without clear spikes were rejected from analyses. NFM jiggle is a measure of the shape variability of consecutive NFMs of an MUPT expressed as a percentage of the total NFM area. NFM segment jitter is a measure of the temporal variability of individual fibre contributions to the NFMs of a MUPT. It is calculated as a weighted average of the absolute values of the temporal offsets between matched NFM segments of consecutive isolated (ie, not contaminated by the activity of other MUs) NFMs across an MUPT expressed in microseconds. NFM dispersion is the time, in ms, between the first and last MU fibre contributions.^21^

### 4.8 Motor unit number estimates

The MUNE value was derived by dividing the negative peak area of the ensemble averaged mean surface MUP (msMUP) from 25% MVC into the negative peak area of the CMAP.^44^ An msMUP is an ensemble average of the negative‐peak onset aligned, sMUPs of the MUs sampled from a muscle. The negative peak area of the msMUP was divided into the negative peak area of the electrically evoked CMAP.^45^ MUNE values are available for 15 males and 15 females.

### 4.9 Statistical analysis

All of the statistical analysis was performed using RStudio (Version 1.3.959 for macOS).^46^ Descriptive statistics of participant characteristics are presented as *mean* ± standard *deviation* (SD). *Student's unpaired t‐test* was used to compare physical parameters (age, BMI, MVC and CSA). As multiple MUs were recorded from each participant, *multi‐level mixed‐effect linear regression analysis* was performed to investigate these MU parameters as well as force steadiness with sex and contraction level as factors through the package *lme4* (Version 1.1.23).^47^ In the linear mixed models, the first level was single motor unit; single motor units were clustered according to each participant to form the second level, which was defined as the participant level. This linear mixed‐effect modelling framework is suitable for data of this nature as it: (i) incorporates the whole sample of extracted MUs not just the mean values obtained from each participant, which preserves variability within and across participants simultaneously to the greatest extent; (ii) handles missing data better than an *analysis of variances (ANOVA)* framework as the removal of a single missing observation has a much smaller effect in the mixed model; and (iii) provides coefficient estimates that indicate the magnitude and direction of the effects of interest.^48^ Interactions were first examined and where not present they were removed from the model, sex and contraction level were explored individually. The results are displayed as coefficient estimates, 95% confidence intervals and *P*‐values. Standardized estimates were calculated through the package *effectsize* (Version 0.4.5)^49^ for forest plotting. For data visualization, individual participant means and group means were shown in box‐and‐jitter plots. Statistical significance was assumed when *P* < .05. Based on the models used, *P* values close to .05 were also addressed.^50^

## CONFLICT OF INTEREST

The authors have no conflict of interest to declare.

## AUTHOR CONTRIBUTIONS

All authors contributed to the conception and design of the work. YG, EJJ, TBI, IAE, JP and MP contributed to the acquisition and analysis of the data. YG analysed the data and drafted the manuscript. BEP, PJA, DJW, KS, DWS, and MP provided comments. All authors have approved the final version of the submitted manuscript for publication and are accountable for all aspects of the work. All persons designated as authors qualify for authorship, and all those who qualify for authorship are listed.

## ETHICS APPROVAL

This research was approved by the University of Nottingham Faculty of Medicine and Health Sciences Research Ethics Committee (C16122016, 160‐0121, 186‐1812, 103‐1809, 302‐1903) and was conducted between 2019 and 2021 in accordance with the Declaration of Helsinki.

## Data Availability

The datasets generated and analysed during the current study are available from the corresponding author upon reasonable request.

## References

[1] Heckman CJ , Enoka RM . Motor unit. Compr Physiol. 2012;2(4):2629‐2682. 23720261 10.1002/cphy.c100087

[2] Enoka RM , Duchateau J . Rate coding and the control of muscle force. Cold Spring Harb Perspect Med. 2017;7(10):a029702. 28348173 10.1101/cshperspect.a029702 PMC5629984

[3] Allen MD , Stashuk DW , Kimpinski K , Doherty TJ , Hourigan ML , Rice CL . Increased neuromuscular transmission instability and motor unit remodelling with diabetic neuropathy as assessed using novel near fibre motor unit potential parameters. Clin Neurophysiol. 2015;126(4):794‐802. 25240249 10.1016/j.clinph.2014.07.018

[4] Piasecki M , Ireland A , Jones DA , McPhee JS . Age‐dependent motor unit remodelling in human limb muscles. Biogerontology. 2016;17(3):485‐496. 26667009 10.1007/s10522-015-9627-3 PMC4889636

[5] Piasecki M , Ireland A , Piasecki J , et al. Long‐term endurance and power training may facilitate motor unit size expansion to compensate for declining motor unit numbers in older age. Front Physiol. 2019;10:449. 31080415 10.3389/fphys.2019.00449 PMC6497749

[6] Del Vecchio A , Casolo A , Negro F , et al. The increase in muscle force after 4 weeks of strength training is mediated by adaptations in motor unit recruitment and rate coding. J Physiol. 2019;597(7):1873‐1887. 30727028 10.1113/JP277250 PMC6441907

[7] Hannah R , Minshull C , Buckthorpe MW , Folland JP . Explosive neuromuscular performance of males versus females. Exp Physiol. 2012;97(5):618‐629. 22308163 10.1113/expphysiol.2011.063420

[8] Hunter SK . Sex differences in human fatigability: mechanisms and insight to physiological responses. Acta Physiol. 2014;210(4):768‐789. 10.1111/apha.12234 PMC4111134 24433272

[9] Haizlip KM , Harrison BC , Leinwand LA . Sex‐based differences in skeletal muscle kinetics and fiber‐type composition. Physiology. 2015;30(1):30‐39. 25559153 10.1152/physiol.00024.2014 PMC4285578

[10] Ansdell P , Thomas K , Hicks KM , Hunter SK , Howatson G , Goodall S . Physiological sex differences affect the integrative response to exercise: acute and chronic implications. Exp Physiol. 2020;105(12):2007‐2021. 33002256 10.1113/EP088548

[11] Hyer MM , Phillips LL , Neigh GN . Sex differences in synaptic plasticity: hormones and beyond. Front Mol Neurosci. 2018;11:266. 30108482 10.3389/fnmol.2018.00266 PMC6079238

[12] Georgieva SDAK . Sex hormones in neurodegenerative processes and diseases. In: Cellular and Molecular Mechanisms of the Effects of Sex Hormones on the Nervous System. London: IntechOpen; 2018.

[13] Mantilla CB , Stowe JM , Sieck DC , et al. TrkB kinase activity maintains synaptic function and structural integrity at adult neuromuscular junctions. J Appl Physiol. 2014;117(8):910‐920. 25170066 10.1152/japplphysiol.01386.2013 PMC4199990

[14] Ansdell P , Brownstein CG , Škarabot J , et al. Menstrual cycle‐associated modulations in neuromuscular function and fatigability of the knee extensors in eumenorrheic women. J Appl Physiol. 2019;126(6):1701‐1712. 30844334 10.1152/japplphysiol.01041.2018

[15] Cowley ES , Olenick AA , McNulty KL , Ross EZ . “Invisible sportswomen”: the sex data gap in sport and exercise science research. Women Sport Phys Activity J. 2021;29(2):1‐6.

[16] Clark BC , Collier SR , Manini TM , Ploutz‐Snyder LL . Sex differences in muscle fatigability and activation patterns of the human quadriceps femoris. Eur J Appl Physiol. 2005;94(1):196‐206. 15791418 10.1007/s00421-004-1293-0

[17] Bolgla L , Cook N , Hogarth K , Scott J , West C . Trunk and hip electromyographic activity during single leg squat exercises do sex differences exist? Int J Sports Phys Ther. 2014;9(6):756‐764. 25383244 PMC4223285

[18] Jones EJ , Piasecki J , Ireland A , et al. Lifelong exercise is associated with more homogeneous motor unit potential features across deep and superficial areas of vastus lateralis. Geroscience. 2021;43:1555‐1565. 33763775 10.1007/s11357-021-00356-8 PMC8492837

[19] Nösslinger H , Mair E , Toplak H , Hörmann‐Wallner M . Measuring subcutaneous fat thickness using skinfold calipers vs. high‐resolution B‐scan ultrasonography in healthy volunteers: a pilot study. Clin Nutri Open Sci. 2022;41:19‐32.

[20] Christie A , Greig Inglis J , Kamen G , Gabriel DA . Relationships between surface EMG variables and motor unit firing rates. Eur J Appl Physiol. 2009;107(2):177‐185. 19544067 10.1007/s00421-009-1113-7

[21] Piasecki M , Garnés‐Camarena O , Stashuk DW . Near‐fiber electromyography. Clin Neurophysiol. 2021;132(5):1089‐1104. 33774377 10.1016/j.clinph.2021.02.008

[22] Piasecki J , Inns TB , Bass JJ , et al. Influence of sex on the age‐related adaptations of neuromuscular function and motor unit properties in elite masters athletes. J Physiol. 2021;599(1):193‐205. 33006148 10.1113/JP280679

[23] Miller AEJ , MacDougall JD , Tarnopolsky MA , Sale DG . Gender differences in strength and muscle fiber characteristics. Eur J Appl Physiol. 1993;66(3):254‐262. 10.1007/BF00235103 8477683

[24] Jeon Y , Choi J , Kim HJ , Lee H , Lim JY , Choi SJ . Sex‐ and fiber‐type‐related contractile properties in human single muscle fiber. J Exerc Rehabil. 2019;15(4):537‐545. 31523674 10.12965/jer.1938336.168 PMC6732543

[25] Taylor AM , Christou EA , Enoka RM . Multiple features of motor‐unit activity influence force fluctuations during isometric contractions. J Neurophysiol. 2003;90(2):1350‐1361. 12702706 10.1152/jn.00056.2003

[26] Harwood B , Cornett KMD , Edwards DL , Brown RE , Jakobi JM . The effect of tendon vibration on motor unit activity, intermuscular coherence and force steadiness in the elbow flexors of males and females. Acta Physiol. 2014;211(4):597‐608. 10.1111/apha.12319 24888350

[27] Peng Y‐L , Tenan MS , Griffin L . Hip position and sex differences in motor unit firing patterns of the vastus medialis and vastus medialis oblique in healthy individuals. J Appl Physiol. 2018;124(6):1438‐1446. 29420154 10.1152/japplphysiol.00702.2017

[28] Inglis JG , Gabriel DA . Sex differences in the modulation of the motor unit discharge rate leads to reduced force steadiness. Appl Physiol Nutr Metab. 2021;46(9):1065‐1072. 33667116 10.1139/apnm-2020-0953

[29] Tenan MS , Peng Y‐L , Hackney AC , Griffin L . Menstrual cycle mediates vastus medialis and vastus medialis oblique muscle activity. Med Sci Sports Exerc. 2013;45(11):2151‐2157. 23657168 10.1249/MSS.0b013e318299a69d

[30] Christie A , Kamen G . Short‐term training adaptations in maximal motor unit firing rates and afterhyperpolarization duration. Muscle Nerve. 2010;41(5):651‐660. 19941348 10.1002/mus.21539

[31] Inglis JG , Gabriel D . Sex differences in motor unit discharge rates at maximal and submaximal levels of force output. Appl Physiol Nutr Metab. 2020;45:1197‐1207. 32338038 10.1139/apnm-2019-0958

[32] Yao W , Fuglevand RJ , Enoka RM . Motor‐unit synchronization increases EMG amplitude and decreases force steadiness of simulated contractions. J Neurophysiol. 2000;83(1):441‐452. 10634886 10.1152/jn.2000.83.1.441

[33] Enoka RM , Christou EA , Hunter SK , et al. Mechanisms that contribute to differences in motor performance between young and old adults. J Electromyogr Kinesiol. 2003;13(1):1‐12. 12488083 10.1016/s1050-6411(02)00084-6

[34] Farina D , Negro F , Dideriksen JL . The effective neural drive to muscles is the common synaptic input to motor neurons. J Physiol. 2014;592(16):3427‐3441. 24860172 10.1113/jphysiol.2014.273581 PMC4229341

[35] Yoon T , Vanden Noven ML , Nielson KA , Hunter SK . Brain areas associated with force steadiness and intensity during isometric ankle dorsiflexion in men and women. Exp Brain Res. 2014;232(10):3133‐3145. 24903120 10.1007/s00221-014-3976-z PMC4172577

[36] Staron RS , Hagerman FC , Hikida RS , et al. Fiber type composition of the vastus lateralis muscle of young men and women. J Histochem Cytochem. 2000;48(5):623‐629. 10769046 10.1177/002215540004800506

[37] Inglis JG , McIntosh K , Gabriel DA . Neural, biomechanical, and physiological factors involved in sex‐related differences in the maximal rate of isometric torque development. Eur J Appl Physiol. 2017;117(1):17‐26. 27815705 10.1007/s00421-016-3495-7 PMC5306324

[38] Inglis JG , Gabriel DA . Is the “reverse onion skin” phenomenon more prevalent than we thought during intramuscular myoelectric recordings from low to maximal force outputs? Neurosci Lett. 2021;743:135583. 33352279 10.1016/j.neulet.2020.135583

[39] Hourigan ML , McKinnon NB , Johnson M , Rice CL , Stashuk DW , Doherty TJ . Increased motor unit potential shape variability across consecutive motor unit discharges in the tibialis anterior and vastus medialis muscles of healthy older subjects. Clin Neurophysiol. 2015;126(12):2381‐2389. 25727901 10.1016/j.clinph.2015.02.002

[40] Piasecki M , Ireland A , Coulson J , et al. Motor unit number estimates and neuromuscular transmission in the tibialis anterior of master athletes: evidence that athletic older people are not spared from age‐related motor unit remodeling. Physiol Rep. 2016;4(19):e12987. 27694526 10.14814/phy2.12987 PMC5064139

[41] Piasecki M , Ireland A , Stashuk D , Hamilton‐Wright A , Jones DA , McPhee JS . Age‐related neuromuscular changes affecting human vastus lateralis. J Physiol. 2016;594(16):4525‐4536. 26486316 10.1113/JP271087 PMC4983624

[42] Williams JS , MacDonald MJ . Influence of hormonal contraceptives on peripheral vascular function and structure in premenopausal females: a review. Am J Physiol‐Heart Circulat Physiol. 2021;320(1):H77‐H89. 10.1152/ajpheart.00614.2020 33164574

[43] Stashuk DW . Decomposition and quantitative analysis of clinical electromyographic signals. Med Eng Phys. 1999;21(6‐7):389‐404. 10624736 10.1016/s1350-4533(99)00064-8

[44] Brown WF , Strong MJ , Snow R . Methods for estimating numbers of motor units in biceps‐brachialis muscles and losses of motor units with aging. Muscle Nerve. 1988;11(5):423‐432. 3374514 10.1002/mus.880110503

[45] Piasecki M , Ireland A , Piasecki J , Stashuk DW , McPhee JS , Jones DA . The reliability of methods to estimate the number and size of human motor units and their use with large limb muscles. Eur J Appl Physiol. 2018;118(4):767‐775. 29356950 10.1007/s00421-018-3811-5 PMC5843678

[46] RStudio Team . RStudio: Integrated Development for R. Boston, MA: RStudio, PBC; 2020. http://www.rstudio.com/.

[47] Bates DMM , Bolker B , Walker S . Fitting linear mixed‐effects models using lme4. J Stat Softw. 2015;67(1):1‐48.

[48] Brown VA . An introduction to linear mixed‐effects modeling in R. Adv Methods Pract Psychol Sci. 2021;4(1):2515245920960351.

[49] Ben‐Shachar M , Lüdecke D , Makowski D . effectsize: estimation of effect size indices and standardized parameters. J Open Source Softw. 2020;5(56):2815.

[50] Greenland S , Senn SJ , Rothman KJ , et al. Statistical tests, P values, confidence intervals, and power: a guide to misinterpretations. Eur J Epidemiol. 2016;31(4):337‐350. 27209009 10.1007/s10654-016-0149-3 PMC4877414

